# Cardiac vagal activity during in-vivo threat exposure is associated with within-session inhibition of fear and avoidance

**DOI:** 10.1038/s41398-026-04333-7

**Published:** 2026-08-01

**Authors:** Christoph Szeska, Kai Klepzig, Alfons O. Hamm, Mathias Weymar

**Affiliations:** 1 University of Potsdam, Department of Biological Psychology and Affective Science, Karl-Liebknecht-Str. 24-25, 14476 Potsdam, Germany; 2 University of Greifswald, Department of Physiological and Clinical Psychology / Psychotherapy, Franz-Mehring-Strasse 47, 17487 Greifswald, Germany; 3 University Medicine Greifswald, Center for Diagnostic Radiology and Neuroradiology, Functional Imaging Unit, 17489 Greifswald, Germany

**Keywords:** Human behaviour, Predictive markers, Prognostic markers

## Abstract

Laboratory research suggests that the attenuation of defensive responses during threat exposure relies on the inhibition of subcortical defense circuits orchestrated by the prefrontal cortex. This process may be reflected by increased vagal efferent activity on the autonomic nervous system. Here, we tested whether cardiac vagal efferent activity, indicated by vagally-mediated heart rate variability (vmHRV), is associated with reduced defensive responses during in-vivo threat exposure and whether it may serve as a physiological index of multi-level exposure responding. Thirty-three women with excessive fear of spiders underwent standardized exposure, where they first approached a living tarantula (approach phase) and subsequently progressed through stages of increasingly direct interactions with the spider (threat imminence task). Indices of cardiac vagal activity (vmHRV) and overall cardiac autonomic arousal (heart rate) were measured alongside self-reported fear and avoidance behavior (distance to spider, performance in threat imminence task). During approach, self-reported fear increased until it reached a stable ceiling. Concurrent vmHRV leveled accordingly, with greater activity being related to lower fear and reduced avoidance during the subsequent threat imminence task. From the very first minute, cardiac vagal activity was associated with the participants’ ability to physically engage with the fear cue during threat imminence task, irrespective of covariations in self-reported fear. Our results suggest that cardiac vagal activity may represent inhibition of fear and avoidance during exposure to perceived threat. Cardiac vagal activity, indexed by vmHRV, could therefore represent an index of within-session multi-level responding to exposure and a promising target for adjuncts to promote fear reduction.

## Introduction

It is well established that the capacity to inhibit defensive responses is compromised in individuals with anxiety disorders, a mechanism thought to underlie symptoms of excessive fear and avoidance [1–3]. This constrained inhibitory capacity is also implicated in high rates of non-responding and relapse in anxiety disorder’s first-line treatment – cognitive behavioral therapy (CBT) – which aims to trigger inhibitory learning through fear cue exposure [4–8]. Despite the importance of inhibitory processes for exposure-based treatments, clinicians currently lack objective and valid real-time markers of a patient’s capacity for defensive response inhibition [9]. In fact, widely applied markers primarily rely on self-report, such as the Subjective Units of Distress Scale (SUDS) [10]. However, due to a lack of validity as a precise measure of fear-related symptoms [11], the SUDS may only inconsistently predict treatment responding [12, 13]. As a result, mechanistic monitoring and real-time prediction of exposure response, as well as adaptive treatment personalization, is currently critically limited.

Rodent models of fear extinction – the laboratory analog of exposure treatment [14] – offer a mechanistic framework for identifying an objective and valid index of defense inhibition: In these models, successful fear extinction relies on the medial prefrontal cortex (mPFC) exerting top-down inhibition over the central amygdala, which organizes defensive responses by downstream projections [15–17]. Importantly, these projections also target brainstem centers of the vagal/parasympathetic (e.g., nucleus ambiguus, dorsal vagal motor nucleus) and sympathetic system (e.g., ventrolateral medulla) [18–20]. Consequently, changes in autonomic balancing during threat exposure may reflect changes in prefrontal inhibitory control over subcortical defense circuits [18–20]. Heart rate and heart rate variability, i.e., the variation in intervals between heart beats, are indicative of changes in cardiac autonomic balancing: Sympathetic effects increase heart rate and act relatively slowly on the heart. The vagal/parasympathetic system, however, affects heart rate rapidly on the time scale of milliseconds, capable of changing beat-to-beat timing of the heart [21]. As a result, rapid fluctuations in heart rate have been conceived as vagally mediated heart rate variability (vmHRV), which represents an index of cardiac vagal efferent activity.

Building upon this framework, recent evidence indeed suggests that vagal efferent activity, reflected by vmHRV [20, 22], posits an index of prefrontal defense inhibition: In humans, increased resting-state vmHRV correlated with increased mPFC volume [23], mPFC activity [21, 24] and mPFC-amygdala functional connectivity [25]. Consequently, laboratory research showed that higher resting-state vmHRV is associated with greater inhibition of defensive behavior towards extinguished threat cues [26, 27].

Accordingly, first clinical translations demonstrated elevated pre-treatment vmHRV to be related to reduced residual symptoms after long-term CBT in patients with anxiety disorders [28, 29]. However, this research focused on resting-state vmHRV – a trait-like measure of cardiac vagal activity correlating with overall emotion regulation capacity [9]. Thus, it still remains unclear whether cardiac vagal activity during exposure also indexes real-time, within-session fear inhibition/treatment response. Only one study examined changes in vmHRV during actual exposure in patients with panic disorder and agoraphobia and observed a decrease in cardiac vagal activity prior to escape behavior [30]. However, it needs to be tested whether cardiac vagal activity reflects inhibition of excessive fear on multiple levels of expression (i.e., behavior and feelings) during *continued* exposure to intense fear cues.

**Fig. 1:**
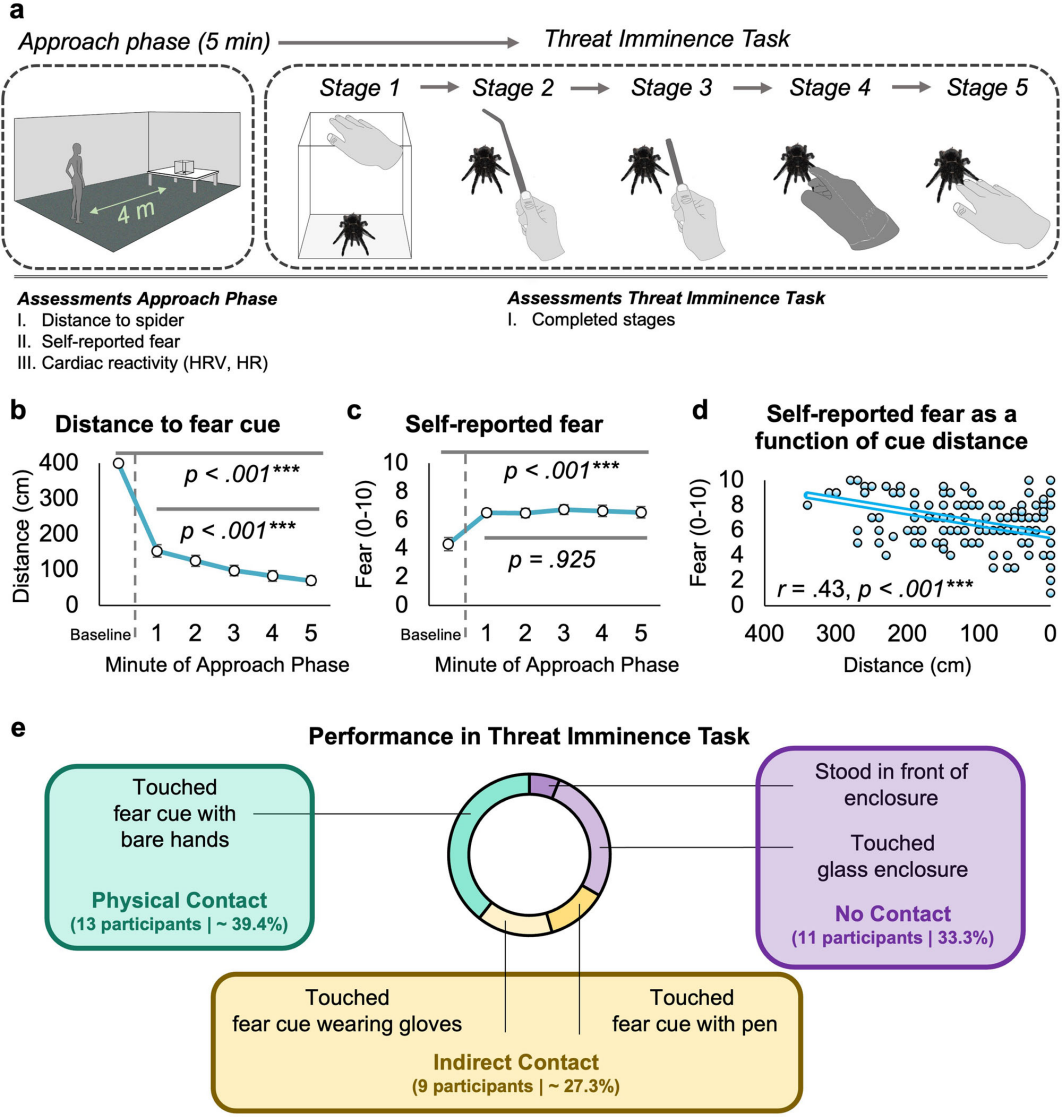
Study design and modulation of defensive response activation during the in-vivo exposure. **a**. Schematic representation of the therapist-guided two-part exposure, starting with the 5-min approach phase, during which distance to tarantula, self-reported fear and cardiac reactivity was assessed. During the following threat imminence task, participants were encouraged to interact with the tarantula in a predefined way: Touch the glass enclosure (stage 1), touch the tarantula using tongs (stage 2), using a short pen (stage 3), wearing gloves (stage 4) and touch the tarantula with bare hands (stage 5). **b** Change in distance to tarantula throughout the approach phase of the in-vivo exposure. **c** Change in self-reported fear throughout the approach phase of the in-vivo exposure. **d** Change in self-reported fear as a function of distance to the fear cue. **e** Distribution of performance in the threat imminence task. Regression lines represent modeled linear relationships. Error bars represent standard error of the mean. *p **<** 0.05, **p **<** 0.01, ***p **<** 0.001.

In the current study, we therefore investigated the potential of cardiac vagal activity as a real-time index of multi-level defense inhibition during exposure, testing its role as a biomarker of dynamic treatment responding. Specifically, we measured parameters of cardiac vagal (vmHRV) and overall cardiac autonomic activity (heart rate) while individuals with excessive fear of spiders underwent a standardized exposure to a living tarantula, which included an initial approach phase and subsequent threat imminence task (see Fig. 1a). This design allowed us to examine within-session changes in cardiac vagal and autonomic activity during the approach phase as a function of critical exposure characteristics (e.g., duration and self-regulated threat proximity). Importantly, we were also able to determine the predictive value of these changes in relation to concurrent variations in self-reported fear and avoidance behavior during the subsequent threat imminence task.

Based on prior preclinical and preliminary clinical research suggesting cardiac vagal activity to reflect defense inhibition in the face of perceived threat [26, 27, 29, 30], we hypothesized that vmHRV serves as an index of real-time responding to exposure, being closely linked to variations in cardinal symptoms of anxiety disorders – excessive fear and avoidance. As a result, (1) higher vmHRV was hypothesized to be associated with lower self-reported fear and increased self-paced approach during fear cue approach phase, and (2) higher vmHRV during such approach was expected to predict physical engagement with the fear cue during the subsequent threat imminence task.

## Material and methods

### Participants

To test our hypotheses, we recruited 33 women who reported a strong fear of spiders from a student sample at the University of Greifswald (*M*_*Age*_ = 22.4, *SD*_*Age*_ = 3.45; *Range* = 18-33; all right-handed). Before enrollment, each participant completed the German version of the Spider Phobia Questionnaire (SPQ; [31, 32]). Individuals were included, who scored one standard deviation or greater above the population mean of the SPQ-15 (i.e., 4.07 ± 3.73) – a subset of fifteen SPQ items, that predict fear and avoidance behavior towards spiders and differentiate between spider-phobic vs. non-phobic individuals [32]. The SPQ-15 sum-score of our sample (*M*_*SPQ-15*_ = 10.69, *SD*_*SPQ-15*_ = 1.40) was comparable to sum-scores reported by spider-phobic patients in previous research (10.67 ± 1.79) [32]. Although no full diagnostic was conducted, participants also completed an additional online questionnaire with items being adapted from the specific phobia section of the DSM-5 (“Diagnostisches Interview für psychische Störungen – DIPS” [33, 34]). With the exception of one individual, all participants (*n* = 32) reported that fear is evoked if they come into contract with a spider or anticipate to do so, that spiders are actively avoided, that fear of spiders has been present for more than six months, and that impairments or burden result from their fear of spiders, indicating phobia-equivalent fear [34].

Furthermore, only students with a body-mass-index between 18.5 kg/m^2^ to 27 kg/m^2^ and an age between 18-35 years were included. The upper BMI limit of 27 kg/m^2^ was selected to also allow the inclusion of individuals in slightly overweight range, who not yet exhibit clinically significant metabolic dysregulation. This approach allowed to effectively increase recruitment feasibility while maintaining a relatively homogeneous sample. The medical and mental status was further controlled by excluding individuals with previous or current conditions that could have affected cardiovascular functions or fear cue processing. To this end, we excluded participants who reported a previous or current cardiovascular, neurological or other bodily condition (e.g., diabetes, hormonal disorders, impaired vision or hearing). Likewise, individuals who reported the wearing of implants (e.g., pacemakers), but also any history of psychotherapeutic treatment, as well as previous or current use of psychotropic drugs were excluded from participation. The applicability of in- and exclusion criteria was assessed on the basis of participants’ self-report. Participants received either course credits or financial compensation for study participation. No individual was excluded from analysis after study participation.

### Design

The standardized two-part exposure took place in a bright and elongated room, in which a glass enclosure containing a docile tarantula (specimen *Tliltocatl albopilosus*; leg span ~ 14 cm) was placed on a table 4 meters in front of the participant and therapist (see Fig. 1a). After entering the room, the exposure started with a five-minute approach phase, during which the participant was encouraged to approach the glass enclosure containing the tarantula as closely as tolerable, starting at the maximum distance of 4 meters. Encouragement (i.e., short validating feedback) was provided by the therapist at predefined points at the beginning and after each minute of the approach phase.

Subsequently, participants were encouraged to sequentially complete different stages in a threat imminence task: Touch the glass enclosure containing the tarantula (stage 1), touch the tarantula using long tongs (stage 2), touch the tarantula using a short pen (stage 3), touch the tarantula while wearing gloves (stage 4) and finally touch the tarantula with bare hands (5). Stages were introduced and demonstrated by the therapist individually. Following a demonstration, participants were given the opportunity to complete the stage, defined as three repetitions of the respective stage criteria (e.g., touch the glass enclosure). Upon successful stage completion, the therapist introduced and demonstrated the subsequent stage, again giving the participant the opportunity of stage completion. There were no time limits for stage completion. If the participant chose not to complete a stage, the task was discontinued. Encouragement was again provided by the therapist at predefined points at the beginning of each stage and after a successful repetition of the stage criteria.

### Assessments and data reduction

#### Approach phase: distance to spider, self-reported fear, heart rate & cardiac vagal activity

Building upon previous research [35], we assessed baseline measures of the participant’s distance to the spider (i.e., 4 m) and self-reported fear at the start of exposure. For the following 5 min of the approach phase, we measured the distance to fear cue in centimeters as well as self-reported fear (measured on a scale ranging from 0 to 10, with 0 representing “no fear” and 10 representing “very severe fear”) on a minute-by-minute basis. Thus, we obtained distance and fear scores for baseline and after 1, 2, 3, 4 and 5 min. The mean distance to the spider, as well as mean self-reported fear was calculated based on distance and fear scores for minutes 1-5. There were no missing data with respect to distance and fear scores.

In addition, cardiac activity was assessed by a Polar H10 chest strap (Polar Electro Oy, Kempele, Finland) in conjunction with the HRV+ Iphone app, which measured the inter-beat intervals (IBI) at a sampling rate of 1000 Hz with high validity [36]. The raw IBIs for the exposure session were analyzed and corrected for both artifacts and very low frequency trend components in Kubios HRV [37] by applying the built-in medium artifact correction and default detrending method (smoothness priors, λ = 500 [38]). For each minute of the approach phase (i.e., after 1, 2, 3, 4, and 5 min) we subsequently determined three indices of cardiac reactivity – two HRV and one HR measure – which all differ in vagal involvement: The root mean square of successive differences (“RMSSD”; in ms) between IBIs refers to a time-domain HRV-measure of cardiac vagal efferent output that is relatively independent of respiratory influences [39, 40]. In contrast, the absolute high frequency power (“HF-Power”; in ms^2^) refers to a frequency-domain (0.15 – 0.4 Hz) HRV-measure of parasympathetic activity that is derived by fast Fourier transformation and is critically shaped by respiratory sinus arrhythmia [39, 40]. Finally, heart rate (in bpm) reflects overall cardiac autonomic output, i.e., the activity of both vagal/parasympathetic and sympathetic branches of the autonomic system [41]. Based on these scores, mean cardiac vagal activity (i.e., RMSSD and HF-power, respectively) and heart rate were calculated by averaging vmHRV and heart rate over minute 1-5 of the approach phase. As in previous research [30] we adjusted for deviations from normal distribution by logarithmically transforming minute- and mean-based HRV data (natural logarithm). There were no missing cardiac data.

#### Threat imminence task: completed stages

Based on the last completed stage in the threat imminence task, the performance of the participant was rated from 0 to 5: 0 = not able to touch the glass enclosure, 1 = touched the glass enclosure containing the tarantula, 2 = touched the tarantula using long tongs, 3 = touched the tarantula using a short pen, 4 = touched the tarantula while wearing gloves, 5 = touched the tarantula with bare hands. Based on these scores participants were further divided into three groups, reflecting the degree of contact with the fear cue: No Contact (i.e., not exceeding stage 1), Indirect Contact (i.e., not exceeding stage 4) and Physical Contact (i.e., completing stage 5). Eleven participants were not able to make contact with the fear cue at all, while nine participants were able to make indirect contact with the tarantula using objects (e.g., by pen or gloves). Thirteen participants were able to make actual physical contact with the fear cue by touching it with their bare hands. There were no missing data with respect to the performance on the threat imminence task.

### Statistical analysis and figure creation

First, we investigated threat-related behavior and feelings throughout the approach phase. To this end, we used generalized least squares (GLS) and modelled changes in distance to the fear cue and self-reported fear as a function of approach phase *Minute* (minutes 0-5 if baseline is included; minutes 1-5 if baseline is excluded). In order to additionally test the relationship between self-reported fear and fear cue distance during the approach phase (minutes 1-5), two-tailed minute-by-minute Pearson correlations between both measures were conducted.

Second, we used GLS to model stage completion during the threat imminence task. In order to additionally test the relationship between average self-reported fear and fear cue distance during the approach phase (minutes 1-5) with stage completion during the threat imminence task, two-tailed Pearson correlations were conducted

Third, we examined cardiac responding throughout the approach phase and tested whether cardiac vagal activity (RMSSD and HF-Power) and heart rate were determined by contextual factors of the exposure (i.e., exposure duration, threat imminence). We therefore used GLS to model changes in cardiac vagal activity and heart rate as a function of *Distance* to the fear cue and approach phase *Minute* (minutes 1-5).

Fourth, we evaluated cardiac responding as a predictor of exposure success from a dimensional perspective. Given our directional a-priori hypothesis that higher cardiac vagal activity is associated with better exposure outcomes, we conducted one-sided Pearson correlations between mean cardiac vagal activity and mean self-reported fear during the approach phase (averaged over minute 1-5 of approach phase), and completed stages during the subsequent threat imminence task (two-sided *p*-values are reported for statistical transparency). Similarly, Pearson correlations between mean heart rate and mean self-reported fear (averaged over minute 1-5 of approach phase) and stage completion (threat imminence task) were conducted. However, as both cardiac deceleration and acceleration are observed depending on perceived threat imminence and avoidance options [42], we decided on a two-sided test.

Finally, we analyzed whether cardiac responding can act as a predictor of exposure success from a categorial perspective. To this end, we used one-sided independent sample *t*-tests to compare mean cardiac vagal activity (approach phase, minute 1-5) between participants of the *Physical Contact* and *No Contact* group. Additionally, to allow for more powerful testing of between-group differences in cardiac vagal activity on a minute-by-minute basis, we modeled cardiac vagal activity as a function of approach phase minute (minutes 1-5) using generalized least squares. Similar analyses were conducted for heart rate, as well. However, as threat can evoke both cardiac deceleration and acceleration [42], the *t*-test was conducted two-sided to compare heart rate between both groups.

Analyses were performed using R [43]. Linear models based on generalized least squares were fitted using the *gls* function of the *nlme* package [44]. In case of minute-by-minute analysis, these models featured a first-order autoregressive covariance structure to account for correlated residuals in repeated measures designs. The significance of effects in these models was tested by *F*-tests using the *anova* function of the *stats* package [43]. Independent-sample *t*-tests were conducted using the *t.test* function [43]. Pearson correlations were computed using the *cor.test* function [43]. The level of statistical significance was set to *p* < 0.05. Tukey corrections were applied to adjust for error inflation in case of multiple post-hoc *t*-tests. Figures were created using Microsoft PowerPoint, Excel and Adobe Illustrator.

An a-posteriori sensitivity analysis using G*Power [45] indicated that linear models based on generalized least squares were capable of detecting medium to large effect sizes (*f*^*2*^ ≥ 0.25). Independent-sample *t-*tests were capable of detecting large effects (*d* ≥ 1.05). Correlational analyses were capable of detecting small to medium effect sizes (*r* ≥ 0.15 for minute-by-minute correlations, *r* ≥ 0.29 for one-tailed correlations between averaged measures, *r* ≥ 0.34 | r ≤ -0.34 for two-tailed correlations between averaged measures).

## Results

### Stable defensive response activation and stable cardiac vagal activity during the in-vivo exposure

During the approach phase of the in-vivo exposure, participants successively closed the distance to the fear cue relative to baseline (*Minute* 0-5, *F*_1, 196_ = 153.26, *p* < 0.001, Fig. 1b), but also across the five minutes of approach (*Minute* 1-5, *F*_1, 163_ = 92.99, *p* < 0.001, Fig. 1b). Concurrently, self-reported fear increased relative to baseline (*Minute* 0-5, *F*_1, 196_ = 18.24, *p* < 0.001, Fig. 1c) and remained stable throughout the following five minutes (*Minute* 1-5, *F*_1, 163_ = 0.09, *p* = 0.925, Fig. 1c), indicating substantial and stable defensive response activation. Importantly, higher self-reported fear corresponded to greater self-regulated distance (i.e., stronger avoidance) to the fear cue on a minute-by-minute basis (minutes 1-5; *r* = 0.43, *p* < 0.001, Fig. 1 d), demonstrating the close relationship between subjective and behavioral fear expressions during the approach (i.e., excluding baseline).

During the following threat imminence task, participants were able to significantly engage with the fear cue (*Intercept, F*_1, 32_ = 99.49, *p* < 0.001, Fig. 1e). Respective performances were largely evenly distributed (Fig. 1e): One third (11 / 33 participants) was not able to make contact with the fear cue at all, while 27% (9 / 33) were able to make indirect contact with the tarantula using objects (e.g., by pen or gloves). Approximately 40% (13 / 33) of the participants were able to make actual physical contact with the fear cue by touching it with their bare hands. Notably, average self-reported fear or average fear cue distance during the approach phase, averaged over minutes 1-5, did not link to the number of completed stages on the subsequent threat imminence task (fear: *r* = -0.09, *p* = 0.618; distance: *r* = -0.16, *p* = 0.347, Supplemental Fig. 1).

**Fig. 2:**
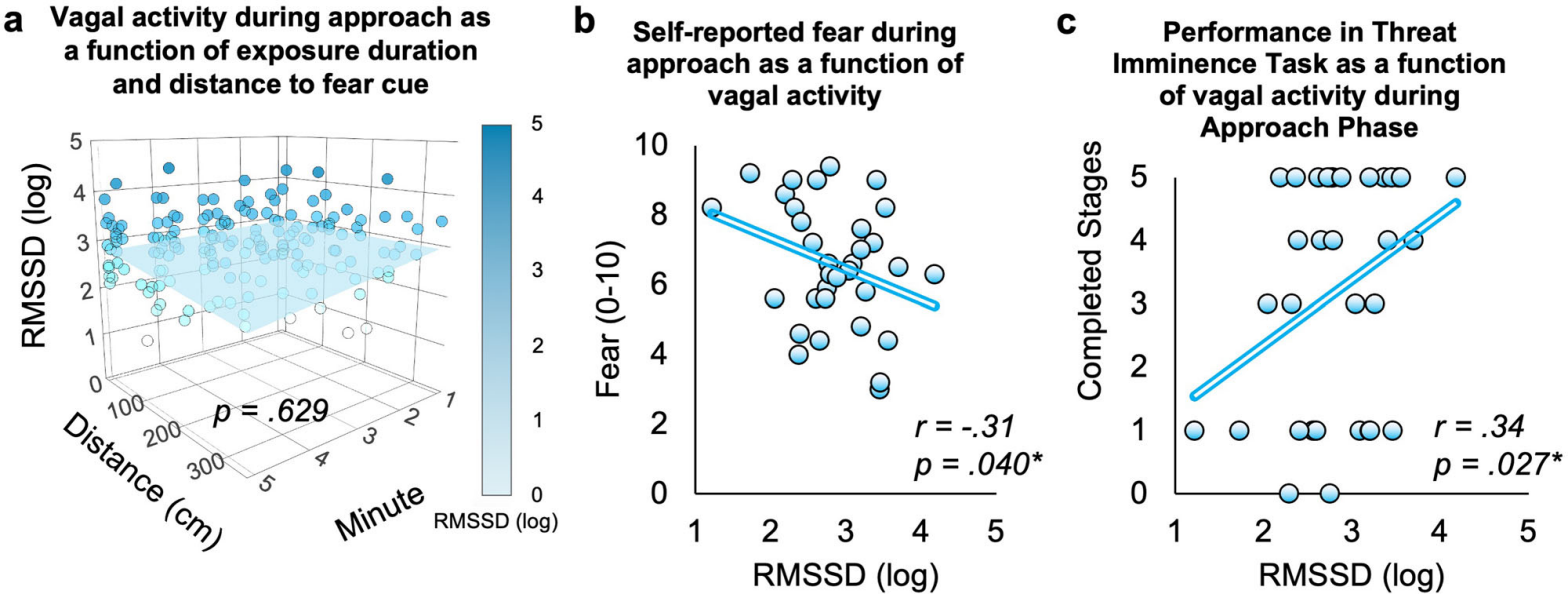
Vagal activity remains stable during in-vivo threat exposure and is associated with inhibition of fear and fear-related behavior. **a** Change in time-domain measure of vagal activity (RMSSD) as a function of both approach phase duration and distance to the fear cue. **b** Scatterplot of average self-reported fear as a function of time-domain vagal activity (RMSSD) during approach phase. **c** Scatterplot of completed stages in the threat imminence task as a function of time-domain vagal activity (RMSSD). Regression lines and planes represent modeled linear relationships. Error bars represent standard error of the mean. *p **<** 0.05, **p **<** 0.01, ***p **<** 0.001.

In addition to changes in defensive response activation, we also tested for changes in cardiac vagal and overall cardiac autonomic activity during the approach phase. Time-domain cardiac vagal activity (RMSSD) remained stable throughout the approach phase (*Minute x Distance*, *F*_1, 161_ = 0.234, *p* = 0.629, Fig. 2a; see also Supplemental Results, Supplemental Fig. 2), which was also observed for the frequency-domain measure of vmHRV (HF-Power; see Supplemental Results, Supplemental Fig. 3). Heart rate, however, significantly habituated throughout exposure (*Minute*, *F*_1, 161_ = 14.330, *p* < 0.001, Supplementary Figure 4a). With regard to the stable cardiac vagal/parasympathetic activity described above, this likely reflected a reduction in sympathetic activity throughout exposure. The distance to the fear cue did not modulate heart rate (*Distance*, *F*_1, 161_ = 1.042, *p* = 0.309, Supplementary Figure 4b; *Minute x Distance*, *F*_1, 161_ = 0.247, *p* = 0.620, Supplementary Figure 4c).

### Cardiac vagal activity is associated with inhibition of fear and fear-related behavior

In the next step, we wanted to investigate how cardiac vagal activity links to defensive response activation throughout the exposure. We found, that higher cardiac vagal activity (RMSSD) was significantly related to lower levels of self-reported fear during the approach phase (*r* = -0.31, one-tailed *p* = 0.040, two-tailed *p* = 0.080, Fig. 2b; for minute-by-minute correlations of RMSSD and fear see Supplemental Figure 5a). In addition, higher average cardiac vagal activity during the approach phase was associated with the completion of more stages in the subsequent threat imminence task (*r* = 0.34, one-tailed *p* = 0.027, two-tailed *p* = 0.057, Fig. 2c). Importantly, these correlations were found to be similar for respiration-dependent frequency-domain measures of cardiac vagal activity (see Supplemental Results, Supplemental Figure 6). Thus, our data suggest that heightened cardiac vagal/parasympathetic activity remains stable during in-vivo exposure and is related to stronger inhibition of fear and fear-related behavior. In contrast, correlations of overall cardiac autonomic activity (heart rate) with self-reported fear or the performance in the threat imminence task was non-significant (all *ps* ≥ 0.159), although the direction of correlations notably mirrored the above described vmHRV findings (see Supplemental Figure 4d, Supplemental Figure 4e, Supplemental Figure 5b).

### Cardiac vagal activity is associated with exposure response early during intervention and independently of fear attenuation

**Fig. 3:**
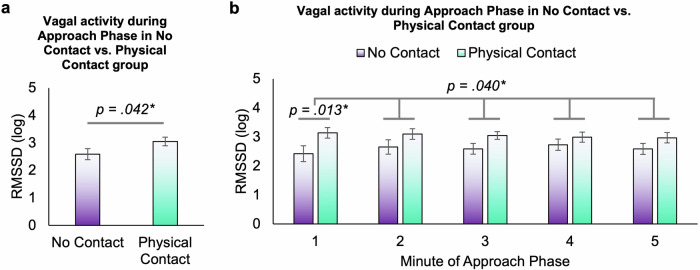
Vagal activity is associated with response to exposure early during intervention. **a** Average time-domain vagal activity (RMSSD) during the approach phase in the No Contact (purple) vs. Physical Contact (green) group. **b** Average time-domain vagal activity (RMSSD) for each minute of the approach phase in the No Contact (purple) vs. Physical Contact (green) group. Error bars represent standard error of the mean. ^(^*^)^*p* < *0.10*, *p < 0.05, **p < 0.01, ***p < 0.001.

Interestingly, increased cardiac vagal activity was not only associated with increased defensive response inhibition from a dimensional perspective, but also represented a categorial predictor of exposure response: During the approach phase, we found significantly higher average cardiac vagal activity in participants who touched the fear cue with bare hands during the subsequent threat imminence task vs. participants who were not able to make any contact (i.e., Physical Contact vs. No Contact Group depicted in Fig. 1e; Group, *t*_*22*_ = 1.810, *p* = 0.042, Fig. 3a). This between-group effect was also significant when analyzing differences on a minute-by-minute basis throughout the approach phase (*Group*, *F*_1, 110_ = 4.318, *p* = 0.040, Fig. 3b), consistent with the notion that vmHRV is linked to subsequent physical engagement with the fear cue. In fact, this effect remained significant even when controlling for self-reported fear as a covariate (*Group*, *F*_1, 100_ = 4.339, *p* = 0.039) indicating that cardiac vagal activity may represent a predictor of exposure response beyond subjective relaxation/attenuated feelings of fear. Importantly, post-hoc tests revealed that the group difference between full- and non-responders was already evident during the first minute of the approach phase (*Group*, *t*_109_ = 2.533, *p* = 0.013, Fig. 3b) suggesting that cardiac vagal activity was associated with exposure response right from the beginning of the session. It needs to be mentioned, however, that we did not find comparably strong and significant group differences for the frequency-domain measure HF-Power (see Supplemental Results, Supplemental Figure 7). Still, we also found higher frequency-domain cardiac vagal activity for the Physical Contact relative to the No Contact group during the first minute of the approach phase (*Group*, *t*_109_ = 2.210, *p* = 0.029, see Supplemental Results for further statistics). Analysis of overall heart rate generally supported the positive relationship between cardiac vagal activity and responding to exposure: We found a trend for lower average heart rate in participants of the Physical Contact, relative to the No Contact group (Group, *t*_*22*_ = 1.759, *p* = 0.092, Supplemental Figure 8a), again consistent with the notion that higher parasympathetic activity is positively associated with responding to exposure interventions. This trend was also observed on a minute-by-minute basis (*Group*, *F*_1, 110_ = 2.849, *p* = 0.094, Supplemental Figure 8b).

## Discussion

Preclinical research highlighted cardiac vagal efferent activity as an index of prefrontal defensive response inhibition during encounters of extinguished threat signals [19–21, 27,46] – a prime mechanism implicated in the effects of exposure-based CBT [5, 14, 47, 48]. Here, we tested whether cardiac vagal activity, reflected by two measures of vagally mediated heart rate variability (vmHRV), could thus serve as an index of exposure response, reflecting within-session changes in excessive fear and avoidance in highly spider-fearful individuals.

We observed stable cardiac vagal activity in the time-domain (RMSSD) while participants approached a fear cue (a tarantula). Here, RMSSD was inversely related to concurrently stable levels of self-reported fear and behavioral avoidance, although these correlations were not robust to two-tailed testing. Additional two-tailed regression models indicated that RMSSD was linked to final physical engagement with the fear cue (i.e., the abolishment of avoidance). Importantly, such predictive value was evident already from the beginning of exposure, irrespective of covariations in self-reported fear. In fact, neither self-reported fear nor self-regulated fear cue distance during initial approach predicted a final physical engagement with the fear cue. Thus, our data are consistent with the notion that time-domain vagal activity may be associated with multi-level exposure responding, explaining more variance in defensive reactivity than self-report or behavioral measures. Frequency-domain measures of cardiac vagal activity (HF-Power) generally mirrored these findings, although the association to behavioral avoidance was less substantial. This might be explained by the stronger sensitivity of HF-Power to respiratory influences relative to RMSSD [49, 50], possibly overshadowing the association between vagal activity and defensive response activation. Finally, heart rate – a compound index of sympathetic and parasympathetic regulation [21] – did not predict self-reported fear or behavioral avoidance, possibly because sympathetic co-regulation distorted comparable associations similar to those observed for vmHRV.

The current results can be interpreted using the Neurovisceral Integration Model [19, 20], which assumes that cardiac vagal efferent activity reflects a homeostatic process, where fluctuations in subcortical defensive response activation (e.g., reflected by fear), are delicately counterbalanced by PFC-mediated inhibition in order to maintain behavioral flexibility [19, 20]. In consequence, cardiac vagal stability may reflect the maintenance of a stable fear state, derived by ongoing defensive response inhibition that allows for adjustments of behavioral approach and avoidance. Indeed, based on the significant positive correlation between self-paced approach and self-reported fear that we observed in the current study, we can assume that participants actively regulated the distance to the fear cue to regulate individual fear. In this framework, decreases in fear, e.g., due to habituation, would be counterbalanced by increased approach to maintain fear at the tolerance threshold. Upholding such “sweet spot” would enable sustained exposure and successive approach to the fear cue at stable concurrent fear levels – as observed in the current study – while effectively preventing escalation of defensive responding (e.g., fight or flight). If cardiac vagal activity indicates this process, as suggested by the Neurovisceral Integration Model [19, 20], higher cardiac vagal activity should consequently be associated with reduced fear, while being decoupled from simultaneous behavioral approach. In addition, stability in self-reported fear would be mirrored by cardiac vagal stability. Indeed, this pattern of results was observed in the current study, with stable cardiac vagal activity being inversely related to concurrently stable fear, while being decoupled from ongoing approach behavior.

Our data are therefore consistent with previous evidence showing that stronger and more stable parasympathetic output is associated with a reduced likelihood of defensive response escalation during exposure (panic attacks, reactive escape) [30]. This supports the notion that cardiac vagal activity may reflect ongoing (prefrontal) defensive response inhibition. Crucially, however, such ongoing inhibition may reflect not only state-dependent moment-to-moment inhibition, but also the real-time utilization of a trait-like inhibitory capacity. Thus, cardiac vagal activity may have tracked moment-to-moment inhibition of fear and avoidance within an individual, as well as individual’s trait-like capacity to exhibit such defensive response inhibition in the face of a threat. Future studies should include both resting-state measures of cardiac vagal activity and within-session vmHRV during repeated exposures in longitudinal designs to disentangle these components more precisely.

Importantly, though, previous research considered vagal activity not only as an index of defensive response inhibition, but also as an index of the organism’s actual defense stage: Increased parasympathetic output has been considered a hallmark of *post encounter defense* – a defense stage that is evoked upon intermediate levels of threat imminence [51–56]. *Post encounter defense* is defined by moderate levels of fear and increased attentional processing of threat signals, which critically differs from *circa strike defense*, which is evoked at highest threat imminence and is associated with high fear, reduced threat processing and active defense (e.g., escape) [51–56]. Importantly, such *circa strike defense* is also characterized by strong sympathetic dominance, as indicated by cardiac acceleration, supporting active defense behavior [42]. Thus, as formalized in the Threat Imminence and Defense Cascade Model [54, 55], higher vagal/parasympathetic activity may reflect a stage of intermediate defensive response activation, which enables thorough processing of perceived threat cues at moderate levels of fear and involves less pronounced disposition for defensive action. Previous research reported that inhibitory extinction learning – a core principle of exposure [57] – can be facilitated by increasing attention to extinguishing fear cues [58–60], and lower levels of self-reported fear [61] and avoidance tendencies [62] are associated with improved exposure treatment response. In line, we found that higher cardiac vagal activity was associated with reduced concurrent fear, and was linked to final physical engagement with the fear cue already from the first minute of exposure.

Following the strategy of the Research Domain Criteria (RDoC) initiative proposed by the National Institute of Mental Health (NIMH) [63, 64], the current study was guided by neural models of fear extinction [17, 48, 65, 66], Neurovisceral Integration [19, 20], Threat Imminence [54] and Defense Cascade [55] to derive and test an actionable, objective biomarker for exposure response in anxiety disorders [67–69]. Self-report markers of treatment responding may be susceptible to biases (e.g., social desirability [70]) and lack validity as a precise measure of fear-related symptoms [11]. As a result, such markers may inconsistently predict treatment responding [12, 13] or provide clinicians with biased information, potentially limiting therapeutic efficacy. In contrast, an objective marker that taps into the mechanisms of action underlying treatment response may provide more valid information about individual threat processing. Thus, it may better predict and even facilitate therapeutic outcome by enabling efficient therapeutic adjustments. Prior research already proposed a range of such biomarker candidates, including genetic polymorphisms [71, 72], neural substrates (e.g., anterior cingulate cortex function) [69], as well as cardiovascular factors (e.g., adrenoreceptor density) [68]. However, the assessment of these potential biomarkers requires extensive training, costly technology (e.g., fMRI), and may not be conducted during treatments. This study provides preliminary evidence that cardiac vagal activity – as reflected by vmHRV – may represent a physiological correlate of multi-level exposure responding that is easy and cost-efficient to assess. Continuous monitoring of vmHRV during exposure may therefore not only provide valid information about the current state of treatment responding, but could also enable individual adaptation of treatment strategies, improving therapeutic outcome by targeting vagal activity [73].

In sum, the current results suggest that cardiac vagal efferent activity may be associated with defensive response inhibition during the exposure to fear cues. Future research building upon this foundation may open up new avenues for mechanistic monitoring and real-time prediction of exposure response, as well as treatment personalization to optimize therapeutic success in anxiety disorders.

## Limitations

While the current study provides preliminary evidence for the potential of cardiac vagal activity to index within-session exposure responding, several limitations should be noted: From a general methodological perspective, a pre-registration protocol would have made the a-priori selection of tests more transparent. From a psychophysiological perspective, it is important to note that we only assessed cardiac responding via a Polar H10 chest-strap. Although highly correlated to multi-lead electrocardiograms (ECG) [36] an ECG would have provided even higher data accuracy. In addition, we did not assess respiration patterns alongside cardiac measurements. However, heart rate variability – and HF-Power in particular – are markedly influenced by respiratory patterns [49, 50], and thus vmHRV in the current study may have captured phasic/respiration-dependent, phasic/state-dependent and tonic vagal activity. Third, we did not assess any information regarding the menstrual cycle of our participants. However, recent work suggested hormonal changes across the menstrual cycle, with lower vmHRV during the luteal phase in women with high premenstrual symptoms [74, 75]. Finally, we did not assess any resting state vmHRV, limiting our ability to fully disentangle trait-like and context-specific regulatory capacity as reflected by vmHRV. Future research should therefore investigate whether the current results can be replicated when using an ECG for vmHRV calculation, taking into account respiration, menstrual cycle and resting state vmHRV as potential confounding covariates.

Most importantly, however, the generalizability of our results is limited: The small sample size limits the statistical power of the analyses, and our a-posteriori sensitivity analysis indeed indicated that small effects might have gone undetected. In addition, correlations of cardiac vagal activity with fear and avoidance were not robust to two-tailed testing and should therefore be considered preliminary. Moreover, the current sample only comprised non-clinical female participants with a similar social status, who underwent a single, rather short, and highly standardized exposure session. While this adds to the internal validity of the study – a prerequisite for a proof of concept that cardiac vagal activity may serve as an actionable biomarker of exposure response – it compromises its external validity. Therefore, it is unclear whether cardiac vagal activity also serves as an index of real-time exposure responding in large and more heterogeneous, clinical samples. Likewise, it remains elusive whether such indicative value covaries with changes in exposure conditions (e.g., changes in exposure contexts, level of guidance by therapists), exposure frequency, exposure duration and targeted exposure mechanisms (e.g., habituation, extinction, deepened extinction or counter-conditioning). In fact, self-reported levels of fear and avoidance tendencies are strongly influenced by these factors and thus cardiac vagal activity may covary alongside [57]. Notably, there is already evidence that the course of vmHRV during an exposure predicts the occurrence of panic attacks and escape behavior in patients with panic disorder and agoraphobia [30]. Current findings therefore are consistent with the notion that cardiac vagal activity could represent a real-time index of treatment responding. Nevertheless, future research needs to address the above-mentioned limitations before cardiac vagal activity can be considered a definitive biomarker of exposure response.

## Supplementary information

Supplemental Results

## Data Availability

Data and code, that was used in this study, is available upon request from the corresponding author.
