# Supplementary material for: Cardiac vagal activity during in-vivo threat exposure is associated with within-session inhibition of fear and avoidance
Source: Transl Psychiatry. 2026 Aug 1;16:388. doi: 10.1038/s41398-026-04333-7 (PMC13428751; doi:10.1038/s41398-026-04333-7)
Supplement: Supplementary file 1 — Supplemental Results [file 41398_2026_4333_MOESM1_ESM.pdf]

Supplemental Results

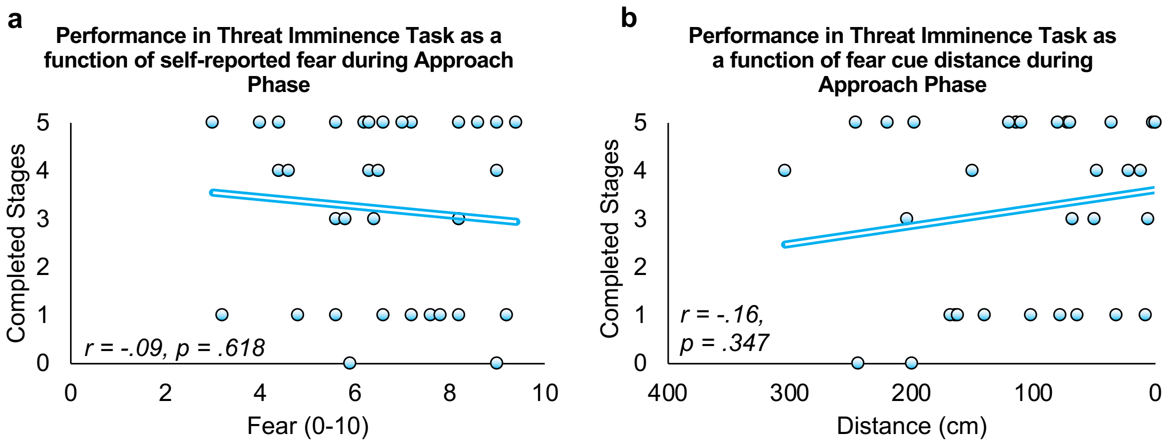

Supplemental Figure 1. Self-reported fear and fear cue distance during approach phase do not predict subsequent performance on Threat Imminence Task.

(a) Scatterplot of completed stages in the threat imminence task as a function of average self-reported fear during the approach phase. **(b)** Scatterplot of completed stages in the threat imminence task as a function of average fear cue distance during the approach phase. Regression lines represent modeled linear relationships.

Time- and frequency-domain measures of vagal activity (HF-Power) remain stable during in-vivo exposure

Throughout the approach phase of the exposure, cardiac vagal activity (RMSSD) remained stable across time (Minute, $F_{1, 161}$ = 0.195, p = .659, **Supplemental Figure 2a**) and varying distance to the fear cue (Distance, $F_{1, 161}$ = 0.006, p = .981, **Supplemental Figure 2b**).

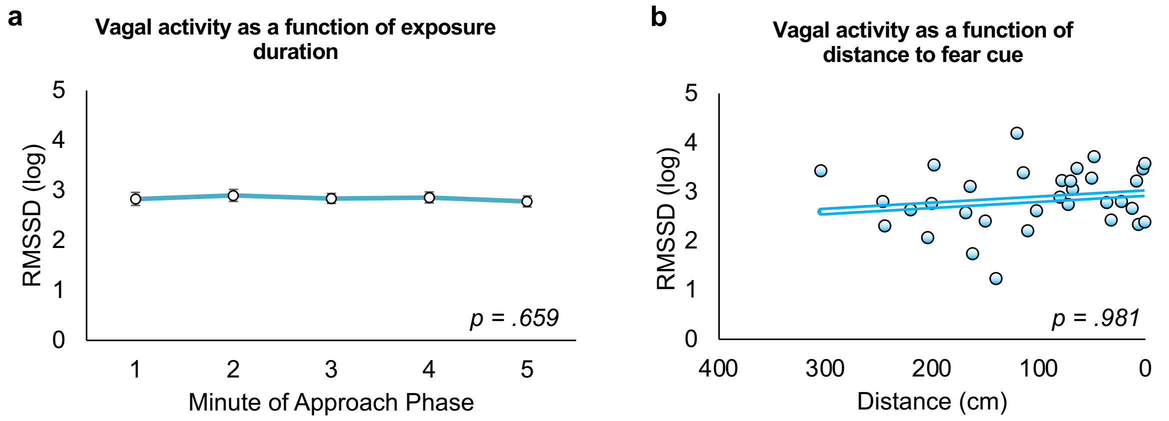

Supplemental Figure 2. **Time-domain measure of vagal activity (RMSSD) remains stable during in-vivo threat exposure.**

(a) Change in time-domain measure of vagal activity (RMSSD) as a function of exposure duration. **(b)** Change in time-domain measure of vagal activity (RMSSD) as a function of distance to fear cue. Regression lines represent modeled linear relationships. Error bars represent standard error of the mean. *p < .05, **p < .01, ***p < .001.

Likewise, frequency-domain cardiac vagal activity (HF-Power) remained stable (*Minute*, $F_{1,\,161}$ = 0.484, *p* = .488, **Supplemental Figure 3a**) irrespective of the distance to the fear cue (*Distance*, $F_{1,\,161}$ = 2.665, *p* = .105, **Supplemental Figure 3b**; *Minute x Distance*, $F_{1,\,161}$ = 0.071, *p* = .791, **Supplemental Figure 3c**).

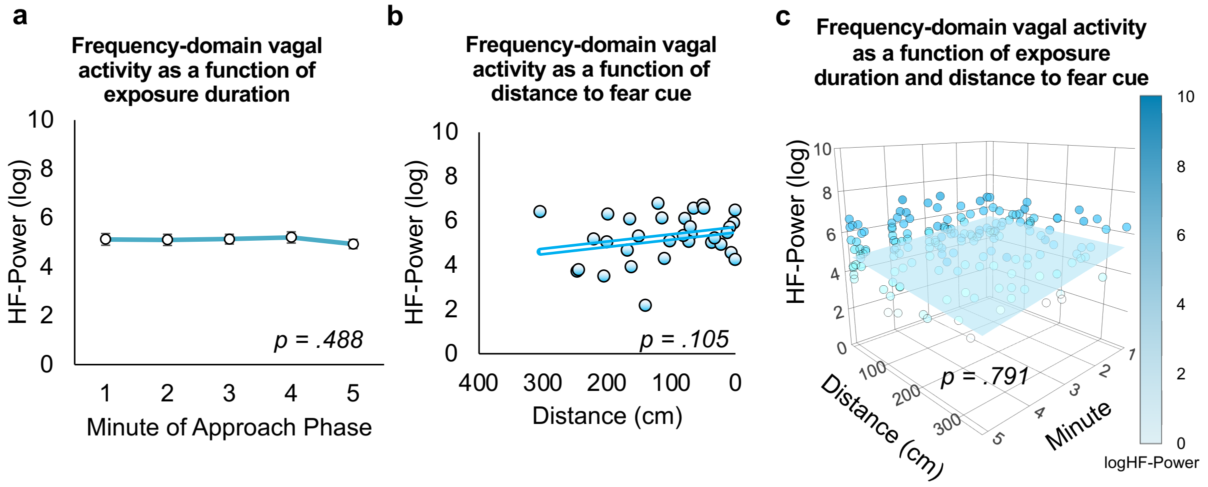

Supplemental Figure 3. Frequency-domain measure of cardiac vagal activity (HF-Power) remains stable during in-vivo threat exposure. (a) Change in HF-Power as a function of exposure duration. **(b)** Change in HF-Power as a function of distance to fear cue. **(c)** Change in HF-Power as a function of both exposure duration and distance to the fear cue. Regression lines and planes represent modeled linear relationships.

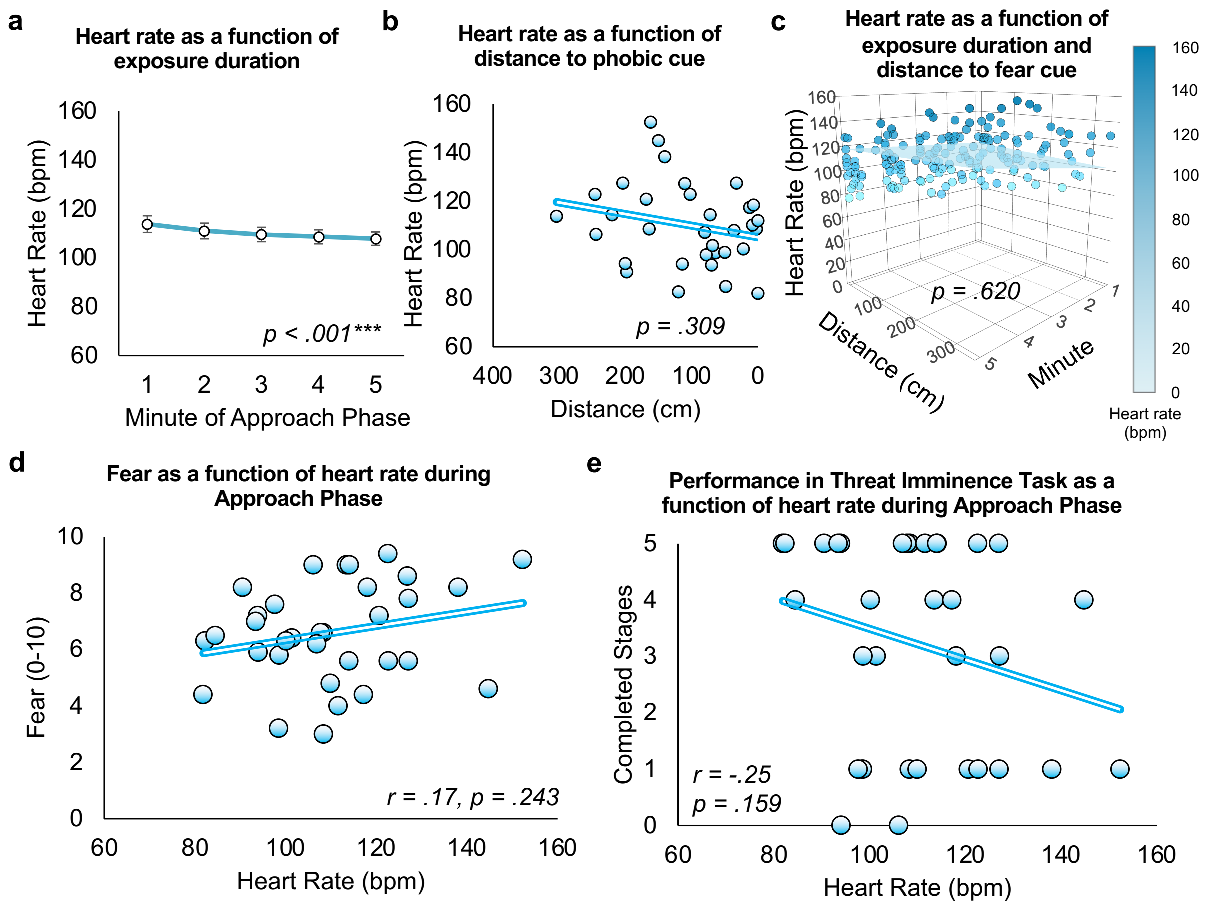

Supplemental Figure 4. Overall cardiac autonomic activity (heart rate) habituates during in-vivo threat exposure and is not associated with fear and fear-related behavior. (a) Change in overall cardiac autonomic activity (heart rate) as a function of exposure duration**.** (**b**) Change in overall cardiac autonomic activity (heart rate) as a function of distance to fear cue**. (c)** Change in overall cardiac autonomic activity (heart rate) as a function of both exposure duration and distance to fear cue. **(d)** Scatterplot of average self-reported fear as a function of overall cardiac autonomic activity (heart rate)**. (e)** Scatterplot of completed stages in the threat imminence task as a function of overall cardiac autonomic activity (heart rate)**.** Regression lines and planes represent modeled linear relationships. Error bars represent standard error of the mean. *$p < .05$, **$p < .01$, ***$p < .001$.

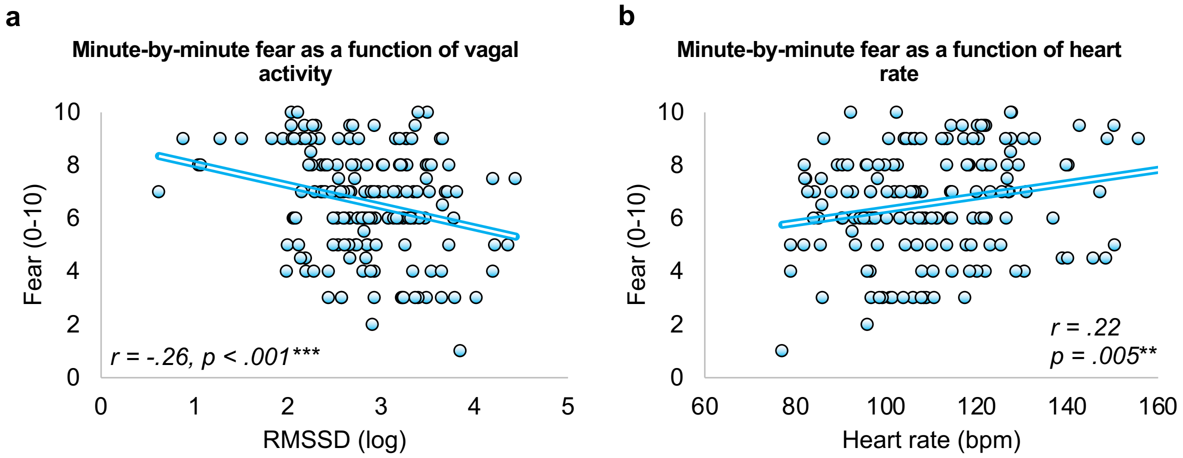

Supplemental Figure 5. Minute-by-minute correlation of cardiac vagal/overall cardiac autonomic activity and self-reported fear. (a) Scatterplot of minute-by-minute self-reported fear as a function of time-domain vagal activity (RMSSD). **(b)** Scatterplot of minute-by-minute self-reported fear as a function of overall cardiac autonomic activity (heart rate). Regression lines represent modeled linear relationships *$p < .05$, **$p < .01$, ***$p < .001$

Frequency-domain measure of vagal activity (HF-Power) is associated with inhibition of fear and fear-related behavior

We found, that higher HF-Power was significantly related to lower levels of self-reported fear during the approach phase (r = -.36, one-tailed p = .021, two-tailed p = .042, **Supplemental Figure 6a**). In addition, we found a trend for higher HF-Power during the approach phase, which was associated with better stage completion in the subsequent threat imminence task (r = .23, one-tailed p = .09, two-tailed p = .18, **Supplemental Figure 6b**).

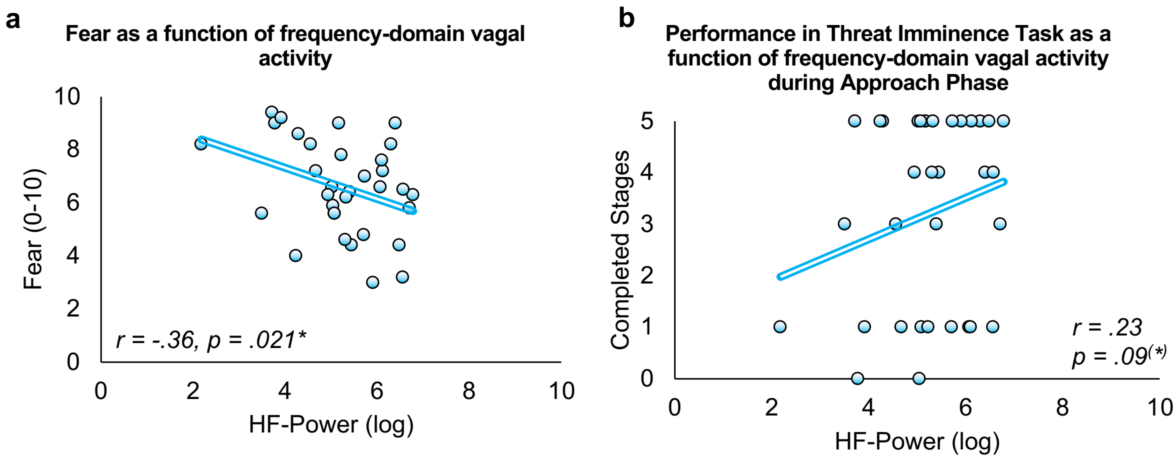

Supplemental Figure 6. Frequency-domain measure of cardiac vagal activity (HF-Power) is related to attenuated fear and fear-related behavior. (a) Scatterplot of average self-reported fear as a function of HF-Power. **(b)** Scatterplot of completed stages in the threat imminence task as a function of HF-Power. $^{(*)}p < .10$, $^{*}p < .05$, $^{**}p < .01$, $^{***}p < .001$.

Frequency-domain measure of vagal activity (HF-Power) is associated with treatment response early during exposure

Participants that were able to make physical contact with the fear cue during the threat imminence task (Physical Contact group), did not show significantly higher frequency-domain vagal activity during the previous approach phase in comparison to individuals of the No Contact group (Group, t_{22} = 1.019, p = .159, **Supplemental Figure 7a**). Correspondingly, we also found no significant between-group effects when analyzing differences in HF-Power on a minute-by-minute basis throughout the exposure (*Group*, $F_{1, 110}$ = 2.066, p = .153, **Supplemental Figure 7b**). Post-hoc tests, however, indicated a significant between-group difference between the Physical Contact vs. No Contact group during the first minute of the session, with higher frequency-domain vagal activity for participants who made final physical contact to the fear cue (*Group*, t_{109} = 2.210, p = .029, **Supplemental Figure 7b**).

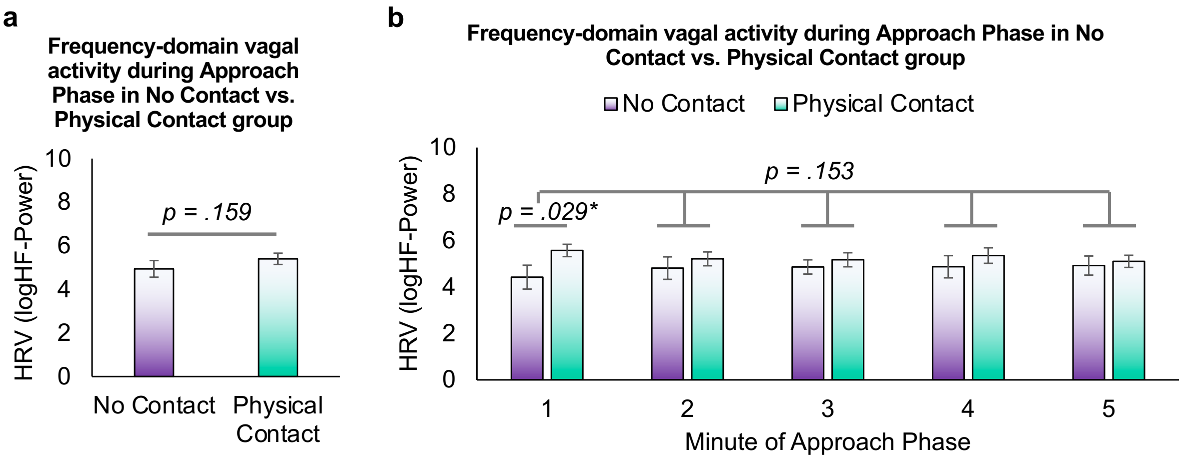

Supplemental Figure 7. Frequency-domain measure of cardiac vagal activity (HF-Power) is associated with response to exposure early during intervention. (a) Average frequency-domain vagal activity (HF-Power) during the approach phase in the No Contact (purple) vs. Physical Contact (green) condition. **(b)** Average frequency-domain vagal activity (HF-Power) for each minute of the approach phase in the No Contact (purple) vs. Physical Contact (green) condition. Error bars represent standard error of the mean. $^{(*)}p < .10$, $*p < .05$, $**p < .01$, $***p < .001$.

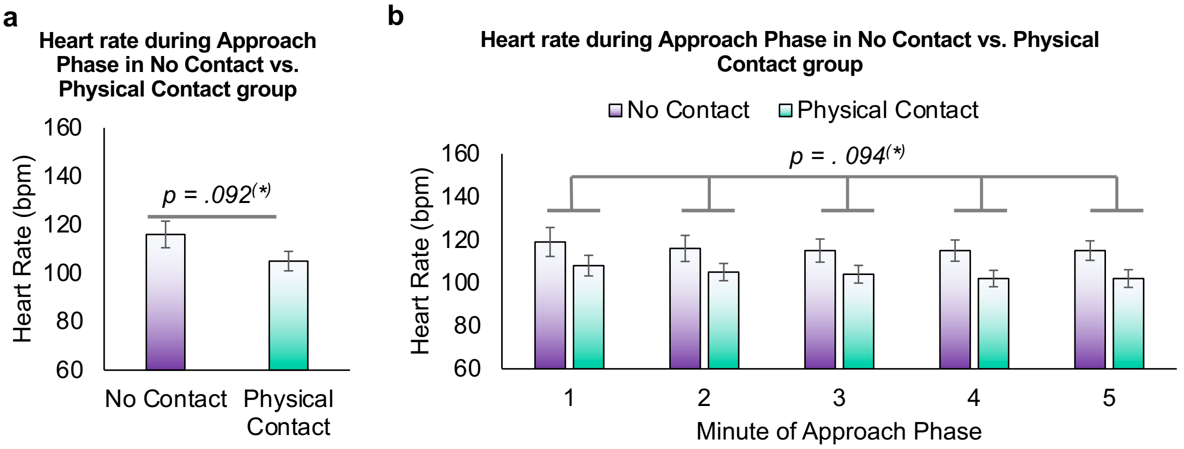

Supplemental Figure 8. Overall autonomic activity (heart rate) does not significantly is associated with response to exposure. (a) Average cardiac autonomic activity (heart rate) during the approach phase in the No Contact (purple) vs. Physical Contact (green) group. **(b)** Average cardiac autonomic activity (heart rate) for each minute of the approach phase in the No Contact (purple) vs. Physical Contact (green) group (d). Error bars represent standard error of the mean. $^{(*)}p < .10$, $^{*}p < .05$, $^{**}p < .01$, $^{***}p < .001$.
